# Efficacy and safety of Xiongzhitongluo granules in the treatment of acute ischemic stroke: study protocol for a randomized controlled trial

**DOI:** 10.3389/fmed.2024.1507278

**Published:** 2025-01-24

**Authors:** Yunmeng Chen, Jingjing Wei, Xiao Liang, Yue Liu, Lina Miao, Di Zhao, Yunfan Zhang, Hongxi Liu, Yunling Zhang

**Affiliations:** ^1^Xiyuan Hospital of China Academy of Chinese Medical Sciences, Beijing, China; ^2^Shenzhen People's Hospital (The Second Clinical Medical College, Jinan University, The First Afiliated Hospital, Southern University of Science and Technology), Shen Zhen, China

**Keywords:** acute ischemic stroke, Xiongzhitongluo granules, randomized controlled study, traditional Chinese medicine, study protocol, efficacy and safety

## Abstract

**Introduction:**

Acute ischemic stroke (AIS) poses a significant risk to human health. Intravenous thrombolysis and mechanical thrombectomy are essential treatments for AIS, offering substantial benefits for neurological recovery and brain protection. However, their efficacy is often limited by stringent time constraints and contraindications, restricting accessibility for certain patient populations. Investigating novel therapeutic strategies is, therefore, crucial. Our team developed Xiongzhitongluo granules specifically for AIS and is conducting a randomized controlled trial (RCT) to validate their effectiveness.

**Methods and analysis:**

This multi-center, randomized, double-blind, placebo-controlled clinical trial includes 120 participants randomly allocated to the intervention or placebo group. Participants will receive a 14-day treatment alongside routine medications and will be monitored at multiple time points: days 1, 3, 5, 7, 14, 30, 60, and 90. The primary outcome is the change in the National Institutes of Health Stroke Scale (NIHSS) score from baseline to day 14. Secondary outcomes include the Scandinavian Stroke Scale (SSS), Barthel Index (BI), modified Rankin Scale (mRS), Brief Mini-Mental State Examination (MMSE), and traditional Chinese medicine (TCM) symptom assessment. Safety evaluations will include vital signs and laboratory tests. Data will be recorded using Epidata V3.1 and analyzed with SPSS 26.0.

**Ethics and dissemination:**

This study received approval from the Ethics Committee of Xiyuan Hospital, China Academy of Chinese Medical Sciences (2021XLA102-2). Written informed consent was obtained from all participants.

**Clinical trial registration:**

https://clinicaltrials.gov/, identifier, ChiCTR2200061859.

## 1 Introduction

Acute ischemic stroke (AIS) is characterized by a sudden interruption of cerebral blood flow, leading to localized ischemia, hypoxic necrosis of brain tissue, and subsequent neurological impairments (1). In China, AIS has become a prevalent non-communicable disease, significantly contributing to mortality and morbidity rates (2–4). Intravenous thrombolysis and mechanical thrombectomy are primary interventions for AIS, offering notable benefits in neurological restoration and brain preservation (5, 6). However, these treatments are often constrained by stringent time requirements and contraindications, limiting their accessibility for certain individuals.

AIS aligns with the traditional Chinese medicine (TCM) concept of “Zhong Feng” (stroke). Previous research has demonstrated the therapeutic potential of TCM in managing AIS, highlighting interventions such as Panax notoginseng saponins (PNS) and Xingnaojing Injection for mitigating cerebral ischemia/reperfusion injury and facilitating stroke rehabilitation (7–9). The integration of Western and TCM approaches in stroke rehabilitation has yielded promising results, suggesting the potential for synergistic outcomes.

Our team’s research identified a strong correlation between AIS episodes and the TCM concepts of blood stasis and toxicity. We hypothesize that the interplay between blood stasis and toxicity, leading to cerebral collateral impairment, represents the fundamental mechanism driving AIS development (10–16). To address this, a standardized system for identifying stasis-toxicity syndromes in the acute phase of cerebral infarction has been implemented (17). Additionally, we proposed a treatment strategy emphasizing blood circulation promotion, stasis dispersion, collateral opening, and toxicity reduction. Building on these findings, our research led to the development of Xiongzhitongluo (XZTL) granules. XZTL granules comprise seven herbs (components detailed in Table 1), each with documented effects on inflammation, cerebrovascular diseases, and related conditions (18–22).

**Table 1:** Components and dose of XZTL granules.

Chinese Pinyin	Latin name	Sources	Weighting (%)*
Chuanxiong	Chuanxiong rhizoma	Ligusticum chuanxiong Hort	17.54
Zhizi	Gardeniae fructus	*Gardenia jasminoides* Ellis	17.54
Sanqi	Notoginseng radix et rhizoma	Panax notoginseng (Burk.) F.H.Chen	10.53
Shuizhi	Hirudo	Whitmania pigra Whitman, OR Hirudo mipponica Whitman, OR Whitmania acranulata Whitmman	8.77
Shichangpu	Acori tatarinowii rhizoma	Acorus tatarinorwii Schott	17.54
Yujin	Curcumae radix	Curcuma wenyujin Y.H, Chenet C. Ling, OR *Curcuma longa* L., OR Curcuma kwangsiensis S.G. Lee et CF. Liang, OR Curcuma phaeocaulis Val.	17.54
Gualou	Trichosanthis fructus	Trichosanthes kirilowii Maxim., OR Trichosanthes rosthornii Harms	26.32

*The weight of every single herb in each bag of XZTL granules (13.86 g).

While individual herbal components have demonstrated efficacy in treating acute cerebral infarction, the effectiveness of their combination remains unclear. To bridge this gap, we designed a multi-center, randomized, double-blind, placebo-controlled clinical trial involving patients with AIS.

## 2 Methods and analysis

### 2.1 Objective

The objective of this study was to evaluate the clinical effectiveness and safety of XZTL granules, offering a novel therapeutic option based on traditional Chinese medicine.

### 2.2 Study design

**Figure 1:**
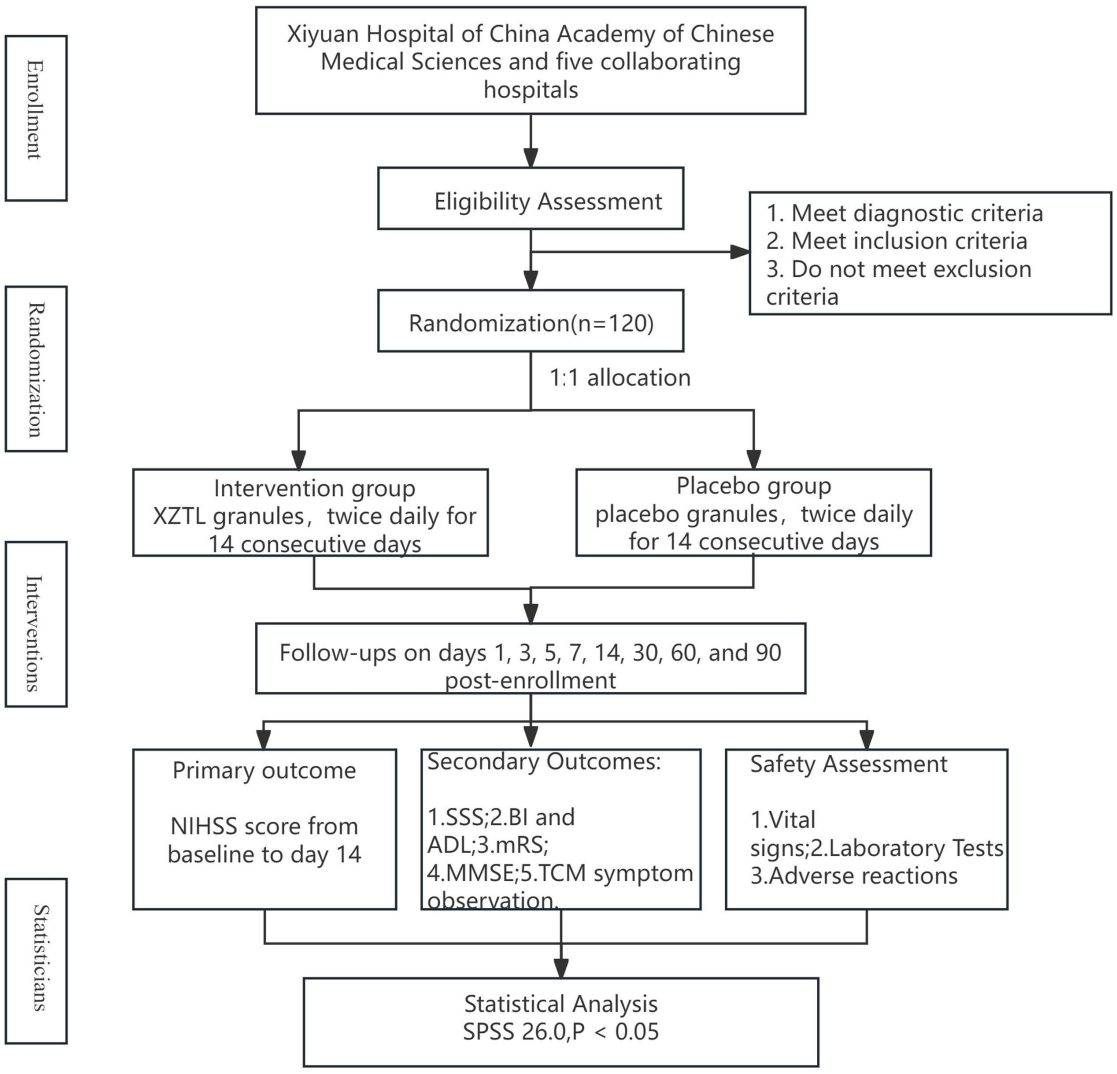
The study design flow chart.

This multi-center, randomized, double-blind, placebo-controlled clinical trial aimed to recruit 120 participants diagnosed with AIS within 72 h of onset from six hospitals (details in Table 2). Participants were randomized in a 1:1 ratio to either the intervention or placebo group. In addition to routine medications, they received 13.86 g of XZTL granules or a placebo in granule form twice daily for 14 days. The study design flow chart is provided in Figure 1.

**Table 2:** The hospitals participating in this study.

Code	Participating hospitals	Area
01	Xiyuan Hospital of China Academy of Traditional Chinese Medicine	Beijing, China
02	Dongfang Hospital of Beijing University of Traditional Chinese Medicine	Beijing, China
03	Affiliated Hospital of Shanxi University of Traditional Chinese Medicine	Shanxi, China
04	Huairou District Hospital of Traditional Chinese Medicine of Beijing Municipality	Beijing, China
05	Chongqing Municipal Hospital of Traditional Chinese Medicine	Chongqing, China
06	Beijing First Integrated Traditional Chinese and Western Medicine Hospital	Beijing, China

This trial was registered with the Chinese Clinical Trial Registry (ChiCTR) under registration number ChiCTR2200061859 and adhered to the Standard Protocol Items: Recommendations for Interventional Trials (SPIRIT) 2013 Statement. Additional details are available in Supplementary material 1.

### 2.3 Eligibility criteria

#### 2.3.1 Diagnostic criteria

##### 2.3.1.1 Western diagnostic criteria

The Western diagnostic criteria for AIS were based on the “Chinese Guidelines for Diagnosis and Treatment of Acute Ischemic Stroke 2018” (23). The criteria included:

(1) Acute onset of the disease.
(2) Presence of focal neurological deficits such as weakness or numbness of one side of the face or limbs, speech disorders, or, in some cases, comprehensive neurological deficits.
(3) Identification of responsible foci on imaging studies or symptoms/signs persisting for more than 24 h.
(4) Exclusion of non-vascular etiologies.
(5) Confirmation of the absence of brain hemorrhage using CT/MRI imaging.

##### 2.3.1.2 Chinese medical diagnostic criteria

The diagnostic criteria for Blood Stasis Toxin Syndrome in AIS were based on the “Diagnostic Criteria for Ischemic Cardiovascular and Cerebrovascular Diseases” (16), as detailed in Table 3.

**Table 3:** Diagnostic criteria for ischemic cardiovascular and cerebrovascular diseases with stasis and toxicity interaction.

Evaluation indicators	Evaluation entries
Required indicators	Persistent, refractory pain or consciousness disorders, accompanied by a rapid and deteriorating clinical course Radiographic findings demonstrate numerous ischemic events or significant vascular lesions, characterized by luminal stenosis of 70% or greater.
Main macro-indicators	Purple tongue or dark purple tongue Sublingual vessel purplish-red or Dark purple Tongue with petechiae or ecchymosis
Secondary macro- indicators	Foul or bitter taste in the mouth. Yellow and dry or yellow and greasy tongue coating. Tough tongue Uneven or wiry pulse.
Laboratory indicators	Elevated C-reactive protein (CRP) levels. Increased platelet aggregation rate. Elevated interleukin-1β (IL-1β). Elevated interleukin-6 (IL-6). Elevated cardiac troponin levels. Elevated fibrinogen levels.

Diagnosis can be established if any of the following criteria are met: ① 1 mandatory indicator plus at least 1 primary macro-indicator, or 2 secondary macro-indicators plus at least 1 laboratory indicator; ② 1 mandatory indicator plus at least 1 primary macro-indicator plus at least 1 secondary macro-indicator.

#### 2.3.2 Inclusion criteria

(1) Fulfilled the diagnostic criteria for AIS.
(2) Met the diagnostic criteria for stroke disease with stasis and toxicity interconnection.
(3) Onset time ≤ 72 h.
(4) National Institutes of Health Stroke Scale (NIHSS) score ≥ 3.
(5) Glasgow Coma Scale (GCS) score ≥ 12 (24).
(6) Age between 18 and 85 years.
(7) Provided informed consent and signed the consent form.

#### 2.3.3 Exclusion criteria

(1) Patients eligible for and prepared to receive thrombolysis.
(2) Patients who had already undergone thrombolytic therapy.
(3) Presence of severe circulatory, respiratory, urinary, or digestive system diseases or cancer.
(4) Abnormal liver or kidney function exceeding two or more times the normal value.
(5) Recent bleeding or bleeding tendency.
(6) Pregnant or lactating women.
(7) Patients with mental disorders who could not cooperate with efficacy assessments.
(8) Patients allergic to any herbal ingredients used in the study protocol.
(9) Participants enrolled in other clinical trials.
(10) Individuals unable to comply with the study protocol.

#### 2.3.4 Termination criteria

(1) The participant’s condition continued to worsen or deteriorated rapidly during the trial, and the attending physician judged that continuation of the clinical trial was inappropriate.
(2) The participant developed comorbidities, complications, or specific physiological changes during the trial that made continued participation unsuitable.
(3) The participant experienced a serious adverse reaction or event during the study period, necessitating withdrawal.

#### 2.3.5 Shedding criteria

Participants were classified as withdrawn under the following circumstances:

(1) Poor compliance with study requirements.
(2) Voluntary withdrawal by the participant.
(3) Failure to adhere to the medication regimen specified in the study protocol.

### 2.4 Sample size calculation

This study was designed as a parallel, multi-center, randomized, double-blind, placebo-controlled trial. The sample size was calculated based on the difference between the NIHSS score for neurological deficits on day 14 of enrollment and the baseline score, which served as the primary efficacy endpoint. The calculation used a test of difference with data from a previous clinical study. In the experimental group, the NIHSS score improved by 4.11 ± 1.34 points, while in the control group, the improvement was 3.42 ± 1.29 points. The test level (*α*) was set at 0.05 (two-sided), with a desired power (1-*β*) of 80%. Based on these parameters, the required sample size for each group was estimated to be 58 participants. To account for potential enrollment deviations in clinical practice, the total planned enrollment was set at 120 participants, with 60 in each group (Figure 1).

### 2.5 Randomization, allocation, and blinding

#### 2.5.1 Randomization and allocation

Randomization was conducted using a block randomization method overseen by statisticians from the Good Clinical Practice (GCP) Center at Xiyuan Hospital, China Academy of Chinese Medical Sciences. The drug coding and randomization table were generated using SAS V9.4 software. Drug codes were assigned from 1 to 160, and the randomization table and scheme were sealed and securely stored at the GCP Center of Xiyuan Hospital.

Before the trial commenced, pre-prepared blinded drugs and corresponding emergency letters were allocated to each center in consecutive number segments. Drug dispensing at each center began with the lowest available number, and drugs were sequentially provided to eligible participants based on their enrollment time.

#### 2.5.2 Blinding

The blinding process was overseen by statistical experts from the GCP Center at Xiyuan Hospital, China Academy of Chinese Medical Sciences. Blinding was conducted by personnel not directly involved in the trial from the randomization and administering units. Initially, blinded personnel verified the consistency of the appearance and packaging of the study drugs across all groups. Subsequently, drug labels corresponding to the assigned drug numbers were affixed to the external packaging of the respective drug groups. The entire blinding process was documented, and records were securely preserved.

Emergency letters containing grouping information corresponding to the drug codes were sealed and distributed to each center along with the pre-prepared blinded drugs. In emergency situations, if the investigator determined it necessary to identify the drug administered for adverse event management, unblinding could be conducted. The unblinding investigator was required to document the reason, date, and signature on the emergency letter upon opening it.

#### 2.5.3 Intervention

All patients enrolled in the study received standard basic treatment as outlined in the “2018 Guidelines for the Diagnosis and Treatment of Acute Ischemic Stroke.” Additionally, participants were randomly assigned to receive either XZTL or placebo granules as an adjunct to standard treatment. The treatment regimen consisted of administering one sachet of granules twice daily for 14 consecutive days.

Both XZTL and placebo granules were produced by Beijing Kangrentang Pharmaceutical Co., Ltd. The placebo was formulated with 95% pure dextrin and 5% of the original herbs used in XZTL, ensuring similarity in properties, odor, color, and appearance to the XZTL granules.

#### 2.5.4 Drugs contraindicated in the study

Participants were required to discontinue the following medications prior to enrollment. Use of these medications during the study was considered a protocol violation:

(1) Chinese medicine injections with the efficacy of activating blood circulation and removing blood stasis or clearing heat and toxins, such as Xuesaitong Injection, Xingnaojing Injection, and Kudiezi Injection.
(2) Proprietary Chinese medicines with the efficacy of activating blood circulation and removing blood stasis, such as Xueshuantong capsules and Naoxintong capsules.
(3) TCM tonics or other preparations with the effect of activating blood circulation, removing blood stasis, detoxifying, or clearing collaterals.

### 2.6 Outcome indicator

#### 2.6.1 Primary outcome

The primary efficacy endpoint of this study was the difference in the National Institutes of Health Stroke Scale (NIHSS) score between baseline and day 14 after enrollment.

#### 2.6.2 Secondary outcome

##### 2.6.2.1 Neurological deficit assessment

The Scandinavian Stroke Scale (SSS) (25) was used to evaluate neurological functions, including consciousness, orientation, eye movements, speech, facial paralysis, upper limb muscle strength, hand muscle strength, lower limb muscle strength, and walking ability. SSS scores range from 0 to 58, with higher scores indicating better neurological status.

##### 2.6.2.2 Daily life ability assessment

The Barthel Index (BI) (26) assessed self-care ability across 10 basic aspects, with a maximum score of 100. Lower BI scores indicated greater incapacity. A BI score > 60 suggested independence with assistance, while a score ≤ 60 indicated dependence on others for daily activities. Additionally, the Ability to Daily Life Scale (ADL) evaluated somatic self-care and instrumental daily life activities, with a total score ranging from 20 to 80. Scores ≤26 were considered normal, while scores >26 indicated varying degrees of functional decline.

##### 2.6.2.3 Disease disability assessment

The modified Rankin Scale (mRS) (26) measured functional independence and disability severity, graded on a scale of 0–6. Higher scores indicated greater disability, while an mRS score ≤ 2 indicated functional independence.

##### 2.6.2.4 Cognitive function evaluation

The Brief Mini-Mental State Examination Scale (MMSE) (27) evaluated cognitive functions, including orientation, recall, attention, calculation, language, and visuospatial abilities. Scores ranged from 0 to 30, with lower scores indicating worse cognitive dysfunction.

##### 2.6.2.5 TCM symptom observation

The TCM Symptom Observation Form for AIS comprised 16 core TCM symptoms, 44 peripheral symptoms, 18 tongue signs, and 12 pulse signs. This multidimensional assessment was conducted by uniformly trained neurologists and incorporated insights from ancient and modern literature, pre-surveys, and expert verification.

### 2.7 Safety assessment

(1) Vital Signs: Temperature, blood pressure, respiration, and heart rate.
(2) Laboratory Tests: Blood routine, seven indicators of liver and kidney function (AST, ALT, GGT, TBIL, BUN, CREA, UA), urine routine, stool routine, coagulation function, and ECG examination.
(3) Adverse reactions/events occurred.

### 2.8 Data management and monitoring

#### 2.8.1 Follow-up program

Visits were conducted on days 1, 3, 5, 7, 14, 30, 60, and 90 post-enrollment. Tasks during visits included data registration, public education, scale assessments, drug distribution, evaluation of efficacy indicators, observation of TCM symptoms, recording of vital signs, and documentation of adverse reactions/events.

Safety evaluations were performed on days 1 and 14 of enrollment. The intervention treatment lasted for 14 days, followed by a follow-up period on days 30, 60, and 90 post-enrollment. The study’s overall flow is summarized in Table 4.

**Table 4:** The overall flow of the study.

Research processes	Visit 1	Visit 2	Visit 3	Visit 4	Visit 5	Visit 6	Visit 7	Visit 8
	Day 1	Day 3	Day 5	Day 7	Day 14	Day 30 ± 3	Day 60 ± 3	Day 90 ± 3
Signed informed consent	√							
Inclusion/exclusion criteria	√							
Demographic information	√							
Medical history	√							
Personal history	√							
Vital signs	√	√	√	√	√	√	√	√
NIHSS score	√	√	√	√	√	√	√	√
SSS score	√	√	√	√	√	√	√	√
GCS score	√	√	√	√	√	√	√	√
mRS score	√	√	√	√	√	√	√	√
Barthel index	√	√	√	√	√	√	√	√
ADL score	√	√	√	√	√	√	√	√
MMSE score	√							√
TCM symptom observation	√	√	√	√	√	√	√	√
Routine blood test	√				√			
Routine Urine	√				√			
Routine stool	√				√			
Liver and kidney function	√				√			
Coagulation function	√				√			
ECG	√				√			
Adverse events	√	√	√	√	√	√	√	√
Consolidation of medication records	√	√	√	√	√	√	√	√
Records of drug dispensing	√							
Record of medication adherence		√	√	√	√			
Records of drug recalls					√			
Completion of trials								√
Statement of audit								√

#### 2.8.2 Data management

The research history form was managed by designated personnel conducting one-on-one assessments based on the observation form’s content. Results were truthfully recorded, and entries were signed for confirmation. Any modifications were marked with a red pen, noting the date and the initials of the modifier, ensuring traceability.

Epidata V3.1 software was used to establish the database. Before data entry, thorough checks of the case history forms ensured completeness, absence of missing pages, accuracy of recorded information, and logical consistency.

Data entry employed a double-entry method to ensure timeliness and accuracy. Computerized audits were conducted during data pre-processing to identify missing values, logical errors, and inconsistencies. Based on audit results, original cases were reviewed, or the data collectors were contacted to verify and ensure traceability of the original data.

Research medical records and related data were sealed and securely stored by the primary research unit.

### 2.9 Statistical analysis

Statistical analyses were performed using SPSS 26.0 software, with two-tailed tests and a significance threshold set at *p* < 0.05. Measurement data were summarized using basic descriptive statistical methods. For normally distributed data, the arithmetic mean and standard deviation were calculated. Intergroup comparisons were conducted using the independent sample t-test, while intragroup comparisons were performed using the paired sample t-test.

For data with a non-normal distribution, the median and interquartile range were calculated, and the non-parametric rank-sum test was employed. Count and rank data were summarized as frequencies and percentages within their respective categories. Statistical analyses of these data sets utilized the chi-square test, Fisher’s exact test, or the Monte Carlo method for exact significance testing.

Analysis of covariance (ANCOVA) was used to evaluate between-group differences in the change from baseline NIHSS scores on day 14. Grouping was included as a fixed factor, and baseline NIHSS scores were used as a covariate. Results were reported as least squares means, standard errors, 95% confidence intervals, *F*-values, and *p*-values.

Repeated-measures analysis of variance (ANOVA) was used to assess changes in repeated-measures data, including neurological deficits, daily living activities, and disease-associated disability levels.

For data that deviated from a normal distribution, a natural logarithmic transformation was applied to achieve approximate normality before statistical analysis. Two-factor ANOVA was performed for data satisfying Mauchly’s sphericity test. Data failing this test underwent Greenhouse–Geisser correction.

Descriptive statistics were applied to analyze the incidence of adverse reactions/events.

## 3 Discussion

The pathological mechanism of AIS is a complex process involving various factors, including blood vessels, cells, and inflammation (28). Occlusion of cerebral blood vessels leads to local ischemia and hypoxia in brain tissues. These conditions disrupt the energy metabolism of nerve cells, resulting in cellular imbalance, degeneration, necrosis, and apoptosis (29). Simultaneously, ischemia- and hypoxia-induced brain tissue damage activates an inflammatory response, triggering the release of inflammatory mediators that exacerbate nerve cell injury (30).

While advancements in intravenous thrombolysis and mechanical thrombectomy techniques have reduced disability and mortality rates in AIS to some extent, their strict time windows and usage restrictions limit their applicability, leaving many patients unable to receive timely and effective treatment. For these patients, who cannot undergo conventional treatments, TCM offers a potential alternative.

XZTL granules, a traditional TCM formulation, have demonstrated multiple therapeutic effects in previous studies, including anti-inflammatory, antiplatelet aggregation, antioxidative stress, and neuroprotective properties. This study aims to evaluate the potential benefits of XZTL granules for AIS treatment through a multicenter, randomized, double-blind, placebo-controlled clinical trial, recruiting patients who have not undergone intravenous thrombolysis or thrombectomy within 72 h of onset.

If the trial results are positive, XZTL granules may provide a new treatment option, offering broader therapeutic opportunities for AIS patients. Furthermore, combining TCM with Western medical treatments may yield more comprehensive therapeutic effects, enhancing recovery rates and improving the quality of life for patients.

## 4 Study status

This study is ongoing and currently in the patient recruitment phase.
