# Supplementary material for: Efficacy and safety of Xiongzhitongluo granules in the treatment of acute ischemic stroke: study protocol for a randomized controlled trial
Source: Front Med (Lausanne). 2025 Jan 24;11:1507278. doi: 10.3389/fmed.2024.1507278 (PMC11802497; doi:10.3389/fmed.2024.1507278)
Supplement: Supplementary file 1 [file Data_Sheet_1.docx]

**TCM Symptom Observation Form**

**Supplementary Table S1 Core Symptom Observation**

| **S/N** | **Symptoms** | **No** | **Yes** |
| --- | --- | --- | --- |
| 1 | Hemilateral paralyze | □ | □ |
| 2 | Numbness in the limbs | □ | □ |
| 3 | Skewed mouth and eyes | □ | □ |
| 4 | Dysphasia | □ | □ |
| 5 | Purple tongue and lips | □ | □ |
| 6 | Petechia or ecchymosis | □ | □ |
| 7 | Gloomy complexion | □ | □ |
| 8 | Tortuous sublingual collaterals | □ | □ |
| 9 | Squamous and dry skin | □ | □ |
| 10 | Restlessness | □ | □ |
| 11 | Red in the face and red in the eyes | □ | □ |
| 12 | Dry mouth, or thick tongue, or dry throat, | □ | □ |
| 13 | Halitosis | □ | □ |
| 14 | sleeplessness | □ | □ |
| 15 | Constipation | □ | □ |
| 16 | Urine with low volume and yellow color | □ | □ |

**Supplementary Table S2 Secondary Symptom Observation**

| **S/N** | **Symptoms** | **No** | **YES** |
| --- | --- | --- | --- |
| 1 | Peak | □ | □ |
| 2 | Obese | □ | □ |
| 3 | Sallow complexion | □ | □ |
| 4 | Pale lips | □ | □ |
| 5 | Tine shakes | □ | □ |
| 6 | Lassitude of spirit | □ | □ |
| 7 | Weak | □ | □ |
| 8 | Breathe hard | □ | □ |
| 9 | Lumbar debility | □ | □ |
| 10 | The head is as heavy as a bundle | □ | □ |
| 11 | Body fatigue, tiredness, and heaviness | □ | □ |
| 12 | Headache | □ | □ |
| 13 | Dizziness | □ | □ |
| 14 | Insomnia | □ | □ |
| 15 | Dreaminess | □ | □ |
| 16 | Hot-tempered | □ | □ |
| 17 | Hot-tempered | □ | □ |
| 18 | Dry eyes | □ | □ |
| 19 | Tinnitus | □ | □ |
| 20 | Bitter taste | □ | □ |
| 21 | Halitosis | □ | □ |
| 22 | Sticky and greasy in mouth | □ | □ |
| 23 | Palpitate | □ | □ |
| 24 | Cough up phlegm cough | □ | □ |
| 25 | Chest tightness | □ | □ |
| 26 | Chest and hypochondriac pain | □ | □ |
| 27 | Frequent deep sighing | □ | □ |
| 28 | Gastric upset | □ | □ |
| 29 | Naxi | □ | □ |
| 30 | Eliminate hunger and hunger | □ | □ |
| 31 | Abdominal distension | □ | □ |
| 32 | Chilliness | □ | □ |
| 33 | Dysphoria in chestpalms-soles | □ | □ |
| 34 | General edema | □ | □ |
| 35 | Spontaneous sweating | □ | □ |
| 36 | Hot flashes and night sweats | □ | □ |
| 37 | Clear abundant urine | □ | □ |
| 38 | Frequent urination at night | □ | □ |
| 39 | Afterurine | □ | □ |
| 40 | Urgency of urination | □ | □ |
| 41 | Weakness in defecation | □ | □ |
| 42 | Loose and thin stool | □ | □ |
| 43 | Poor stool viscosity | □ | □ |
| 44 | Stool irregularity alternating with constipation | □ | □ |

**Supplementary Table S3 Tongue Observation Form**

| **S/N** | **Symptoms** | **No** | **YES** |
| --- | --- | --- | --- |
| 1 | Pale red tongue | □ | □ |
| 2 | Pale tongue | □ | □ |
| 3 | Red tongue | □ | □ |
| 4 | Crimson tongue | □ | □ |
| 5 | Fissured tongue | □ | □ |
| 6 | Enlarged tongue | □ | □ |
| 7 | Thin tongue | □ | □ |
| 8 | Tough tongue | □ | □ |
| 9 | Teeth-marked tongue | □ | □ |
| 10 | Dry mouth | □ | □ |
| 11 | Moist tongue | □ | □ |
| 12 | White tongue | □ | □ |
| 13 | Stiff tongue | □ | □ |
| 14 | Yellow coating | □ | □ |
| 15 | Thin coating | □ | □ |
| 16 | Thick coating | □ | □ |
| 17 | Greasy coating | □ | □ |
| 18 | Little coating | □ | □ |

**Supplementary Table S4 Pulse Evidence Observation**

| **S/N** | **Symptoms** | **No** | **YES** |
| --- | --- | --- | --- |
| 1 | Floating pulse | □ | □ |
| 2 | Deep pulse | □ | □ |
| 3 | Slippery pulse | □ | □ |
| 4 | Neven pulse | □ | □ |
| 5 | Wiry pulse | □ | □ |
| 6 | Tense pulse | □ | □ |
| 7 | Quick pulse | □ | □ |
| 8 | Tardy pulse | □ | □ |
| 9 | Relaxed pulse | □ | □ |
| 10 | Soggy pulse | □ | □ |
| 11 | Thready pulse | □ | □ |
| 12 | Feeble pulse | □ | □ |
